# Ultraviolet aging study on bitumen modified by a composite of clay and fumed silica nanoparticles

**DOI:** 10.1038/s41598-020-68007-0

**Published:** 2020-07-08

**Authors:** Goshtasp Cheraghian, Michael P. Wistuba

**Affiliations:** Braunschweig Pavement Engineering Centre, Technische Universität Braunschweig, Braunschweig, Germany

**Keywords:** Civil engineering, Nanoscale materials, Other nanotechnology

## Abstract

In this study, surface morphology, rheological and chemical properties were investigated of bitumen, which was modified by a composite of clay and fumed silica nanoparticles, and exposed to ultraviolet (UV) aging in laboratory. The volume fraction of the nanoparticles within the binder ranged from 1 to 3%, the temperature range considered was 30 to 70 °C. Surface morphology, rheological and chemical binder properties were analyzed using field emission scanning electron microscopy (FESEM), dynamic shear rheometer (DSR), and Fourier transform infrared (FT-IR) spectroscopy. It was found, that the bitumen modification through clay and fumed silica nanoparticles changed resulting binder properties significantly. The index of carbonyl and oxidation degree decreased, and the clay and fumed silica nanoparticles improved aging resistance to ultraviolet (UV) radiation considerably. The results indicate that the mechanical stability of the modified bitumen is very much driven by the specific concentration of clay and fumed silica nanoparticles.

## Introduction

### Objective of this study

Bitumen is extracted from crude oil. Its composition can be roughly grouped into maltenes (a mixture of saturates + aromatics + resins) and asphaltenes (SARA-fractions)^1–3^. It is well known that aging increases asphaltenes and resins content, while simultaneously decreasing aromatics content^4^, which makes the binder brittle and the asphalt pavement layer vulnerable to cracking^5,6^. Bituminous binders, that are most resistant to aging, are therefore of interest for asphalt pavement engineering.

Oxidation, volatilization, and steric hardening are the most important factors influencing bitumen aging. While steric hardening results from molecular rearrangement, oxidation and volatilization go together with a change of molecules. Temperature is an important factor driving these aging processes, as well as the presence of UV radiation and atmospheric oxygen in regard to oxidation aging. UV radiation may break the bonds of bitumen molecules, and may create free radicals, which in turn accelerate the aging process^7^. Consequently, bitumen modified by anti-aging materials that stimulate anti UV aging properties are of high interest for application in pavement engineering.

In the last decade have been many studies investigating modified bitumen with nanoparticles (NPs)^8–10^. Scientists and researchers are focused on new nanomaterials to meet the improve bitumen properties that conventional bitumen cannot meet these properties^11,12^. However, there are few works related to the effect of NPs on ultraviolet aging properties of bitumen. Titanium dioxide, cuprous oxide, zinc oxide, silicon dioxide, and montmorillonite are some NPs that have been used for improving bitumen aging resistance to UV radiation^13–17^.

The goal of this study was to analyze bitumen modified by a novel anti-aging additive that was composed of clay and fumed silica nanoparticles (CS-NPs; of size in the range of 10–30 nm). Due to their increased ratio of surface area to volume compared to conventional filler particles, CS-NPs enable rich binder contents in asphalt mixtures. Their large surface (20–500 m^2^/g) is a unique character which promotes interaction of particles having a significant effect on the rheological and anti-aging properties of the modified binders^18,19^, also increasing bond strength at the aggregate-bitumen interface^20,21^.

Clay and fumed silica are low-cost nanomaterials, harmless and non-toxic, and they can be used as inorganic shielding^22^. It was reported, that clay and fumed silica nanoparticles (CS-NPs) may potentially improve bitumen mechanical and rheological properties^23–26^, as well as its resistance to moisture damage and aging^27–29^. However, the combination of both of them as a nanocomposite has not yet been used in asphalt mixture. Benefiting from the specific nanostructure of clay nanolayers and fumed silica nanoparticles, the stability^30^ and the reactivity^31^ of nanocomposites may be significantly improved, due to their advantageous physical properties.

This study contributes to the analysis of surface morphology, rheological and chemical binder properties of CS-NPs, in order to present new findings and to strengthen the existing knowledge on using such nanoparticles for bitumen modification.

### Experimental plan

**Figure 1:**
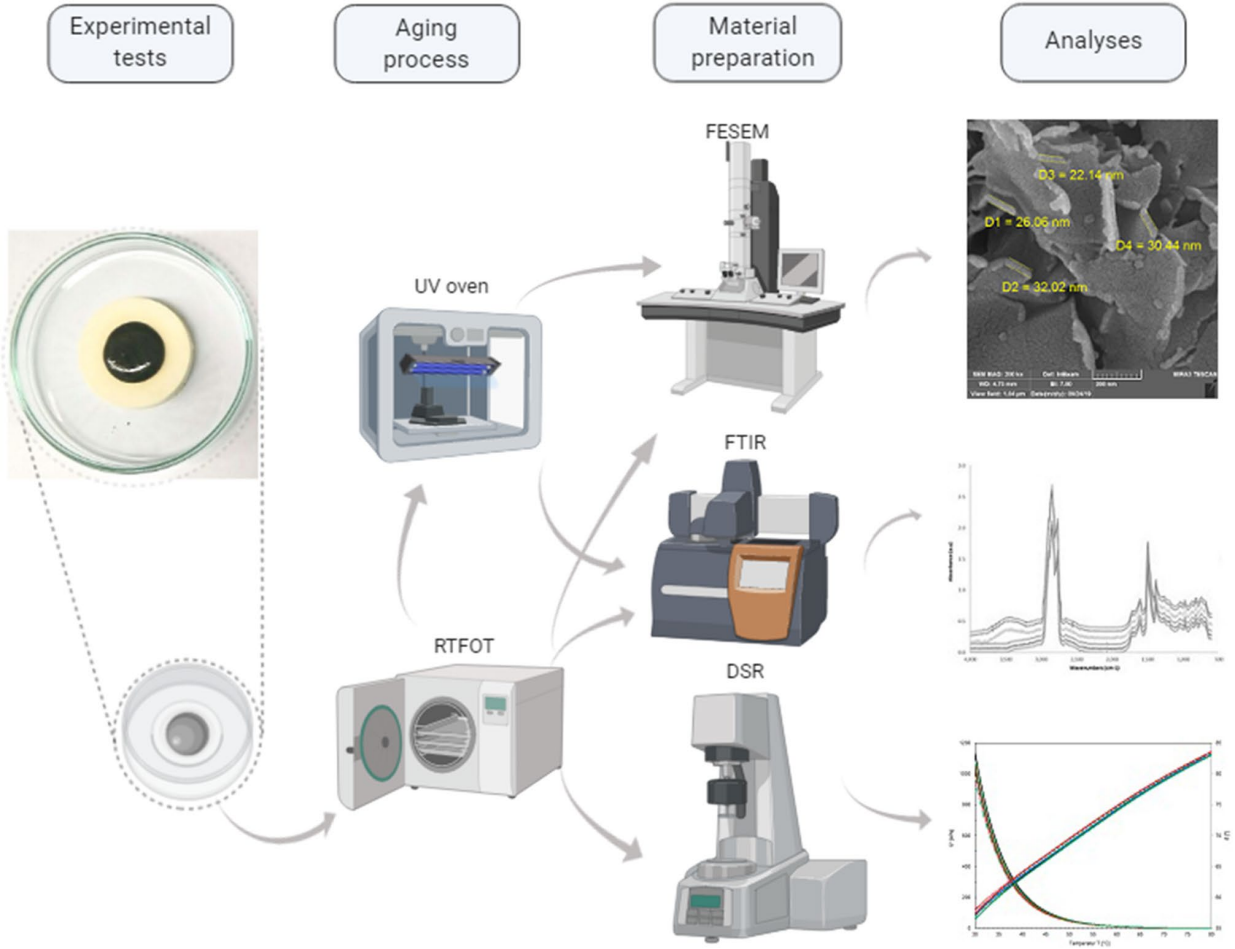
Schematic diagram indicating the material preparation, aging process, and experimental tests. Image partially created with BioRender.

First, surface morphology of clay/fumed silica nanoparticles (CS-NPs) dispersed in the bitumen sample was studied using observations from field emission scanning electron microscopy (FESEM). Then, modified bitumen samples were prepared with different volume fractions of CS-NPs, to study the impact of CS-NPs concentration on resulting binder properties, i.e. rheological binder properties investigated through dynamic shear rheometer (DSR) measurements, and chemical binder properties from Fourier transform infrared (FT-IR) spectroscopy. Information on these test methods is reported in Fig. 1 schematically.

## Results and discussion

### Surface morphology identified with FESEM

**Figure 2:**
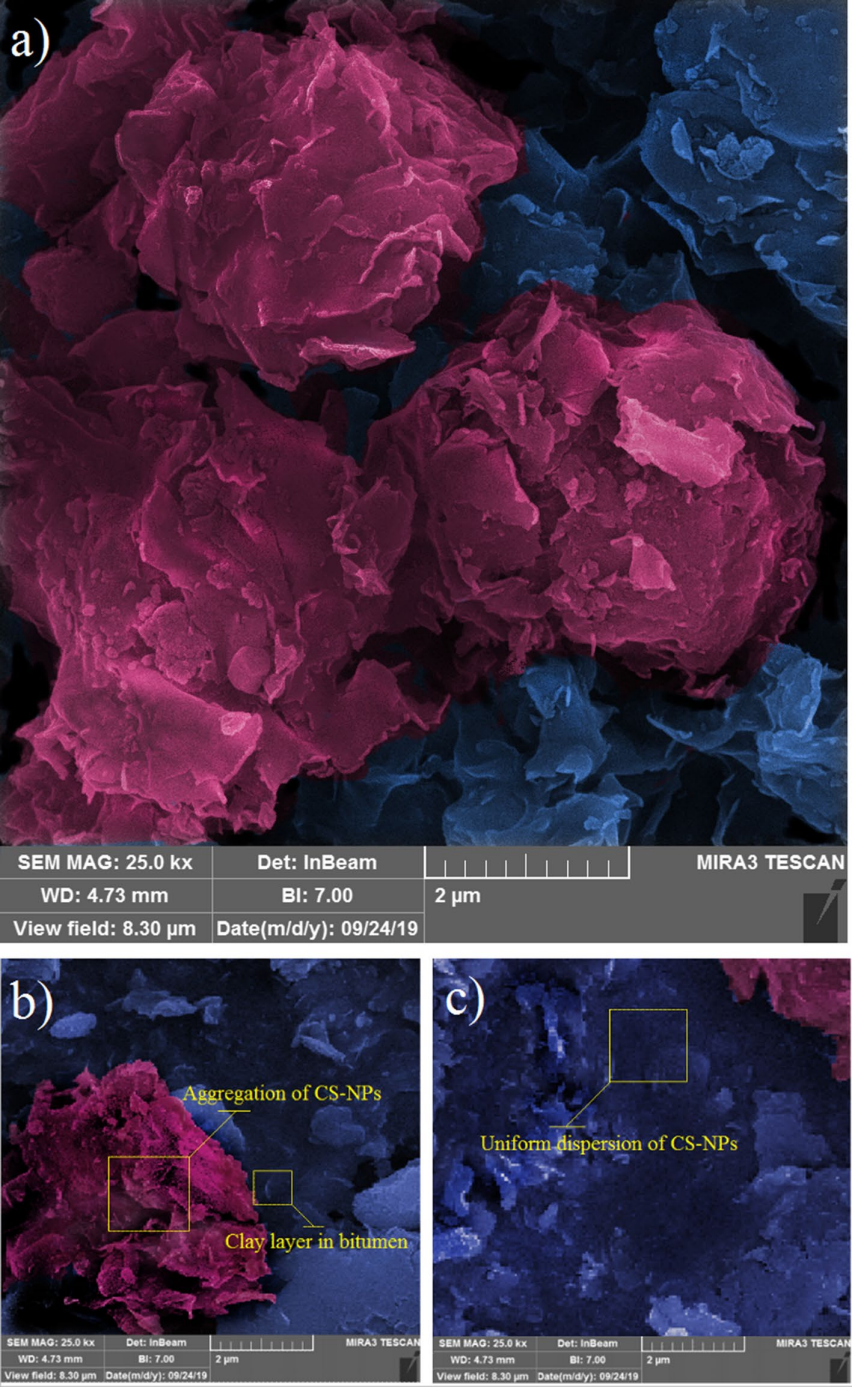
FESEM image of CS-NPs: (**a**) aggregation of clay nanoparticles form a unique chaotic structure of rosebud shape; the blue color represents uniform dispersion of CS-NPs, while aggregation of clay NPs and silica NPs are colored in red; (**b**) CS-NPs aggregation; and (**c**) uniform dispersion of CS-NPs.

Results from FESEM are shown in Fig. 2, illustrating the surface morphology of CS-NPs dispersed in the bitumen sample. A unique complex structure is formed from nanosheets. They look like petals that form structures of rosebud like shape.

Figure 2 uses colors in order to visually separate CS-NPs (colored in blue), and aggregations of CS-NPs (colored in red). The coloring is based on image analysis technique. Initial data were analyzed by a microanalysis software to measure the scale of layers and the diameter of particles. FESEM images were then processed and segmented with an image processing program, for digital analyses, and the topographical result was used as input for a professional coloring software.

When CS-NPs are blended with bitumen, the CS-NPs partially cover the bitumen surface, and when exceeding a specific threshold value the CS-NPs begin to aggregate and create a structure of multiple layers (Fig. 2a). This particle aggregation changes the homogeneity of the blend and may also change its rheological properties. In Fig. 2b, c, red color illustrates the aggregation of CS-NPs. These red sections are considered as challenging parts of bitumen surface and are therefore further investigated in regard to the structure of clay layer with fumed silica NPs. Around the aggregation of CS-NPs, a uniform dispersion zone is seen on the bitumen surface (indicated in blue), which represents bitumen covered by a thin film of CS-NPs (single layers).

The aggregation of clay NPs were found to be uniformly dispersed in the bitumen sample, which indicates that they were uniformly dispersed during the blending process already. The sizes of aggregation of clay NPs range from 400 to 800 nm.

It is assumed, that this chaotic nanostructure in the bitumen, originating from the presence of CS-NPs, prevents the bitumen from aging: on the one hand, it acts like a radiation shield that reflects UV light and preserves the bitumen from UV light penetration. On the other hand, it traps the chemical volatile compounds of bitumen, and it prevents (or at least decelerates) their evaporation.

**Figure 3:**
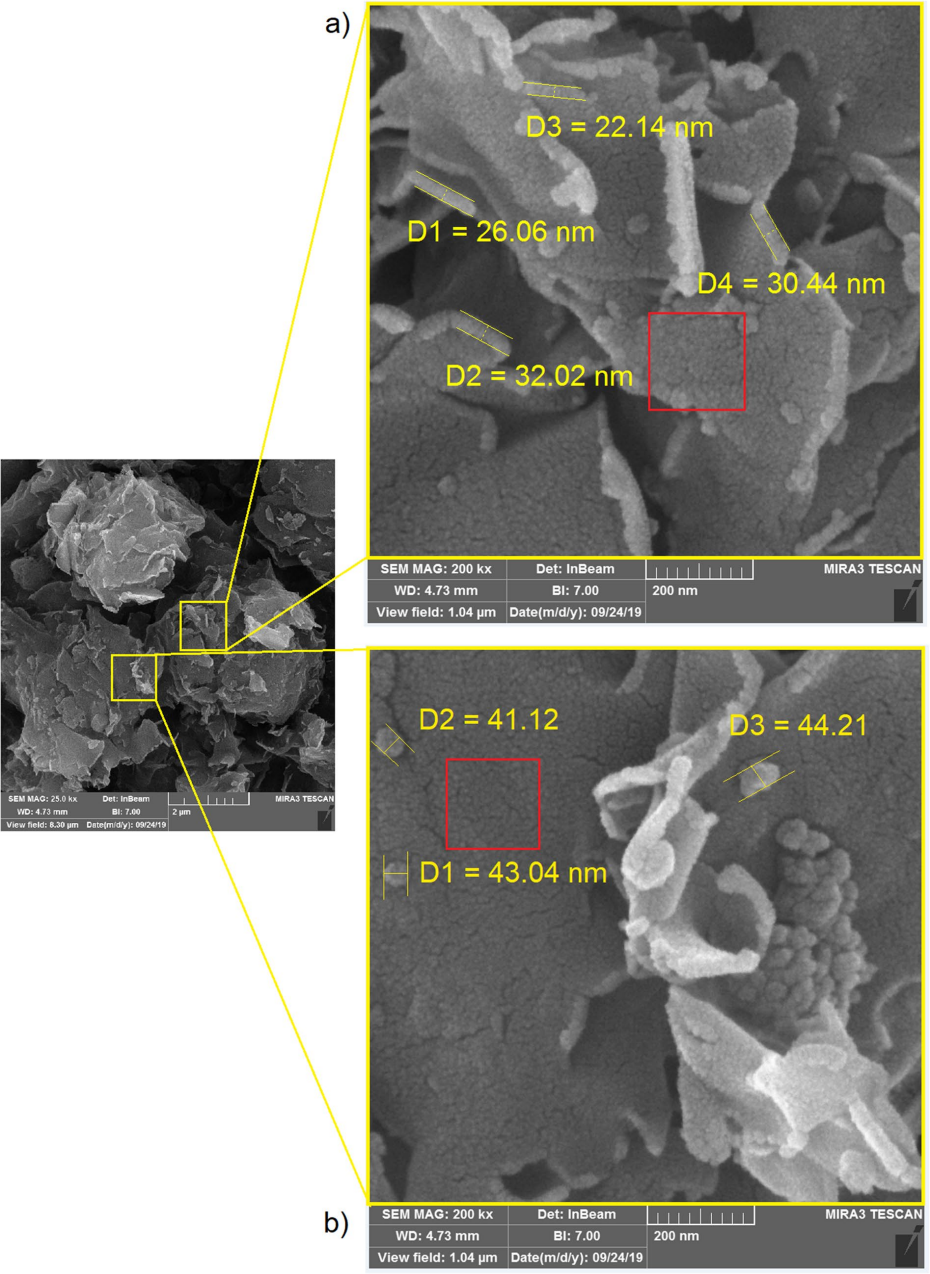
FESEM image of CS-NPs: (**a**) size of clay nanolayers; and (**b**) size of fumed silica nanoparticles.

In Fig. 3 the red colored aggregation of clay NPs and silica NPs is illustrated more in detail, showing that some clay NPs are coated with much smaller silica NPs. Note that some silica NPs form dense bulks on the layers of clay NPs. With the increase of NPs in the surface unit, due to polarity and chemical bonding, nanolayers adsorb together and create bulkily aggregations^32,33^. Contrary, the blue colored structures are (advantageously) bonded with bitumen molecules and they are distributed in bitumen in terms of a uniform surface. As indicated in Fig. 3a, b, the average particle sizes of clay layers are about 27 nm, and the silica NPs are of about 12 nm, respectively. However, the bulk size of aggregation of silica NPs is about 42 nm (Fig. 3b). The red squares in Fig. 3 illustrate that clay layers may entirely be coated by fumed silica NPs.

**Figure 4:**
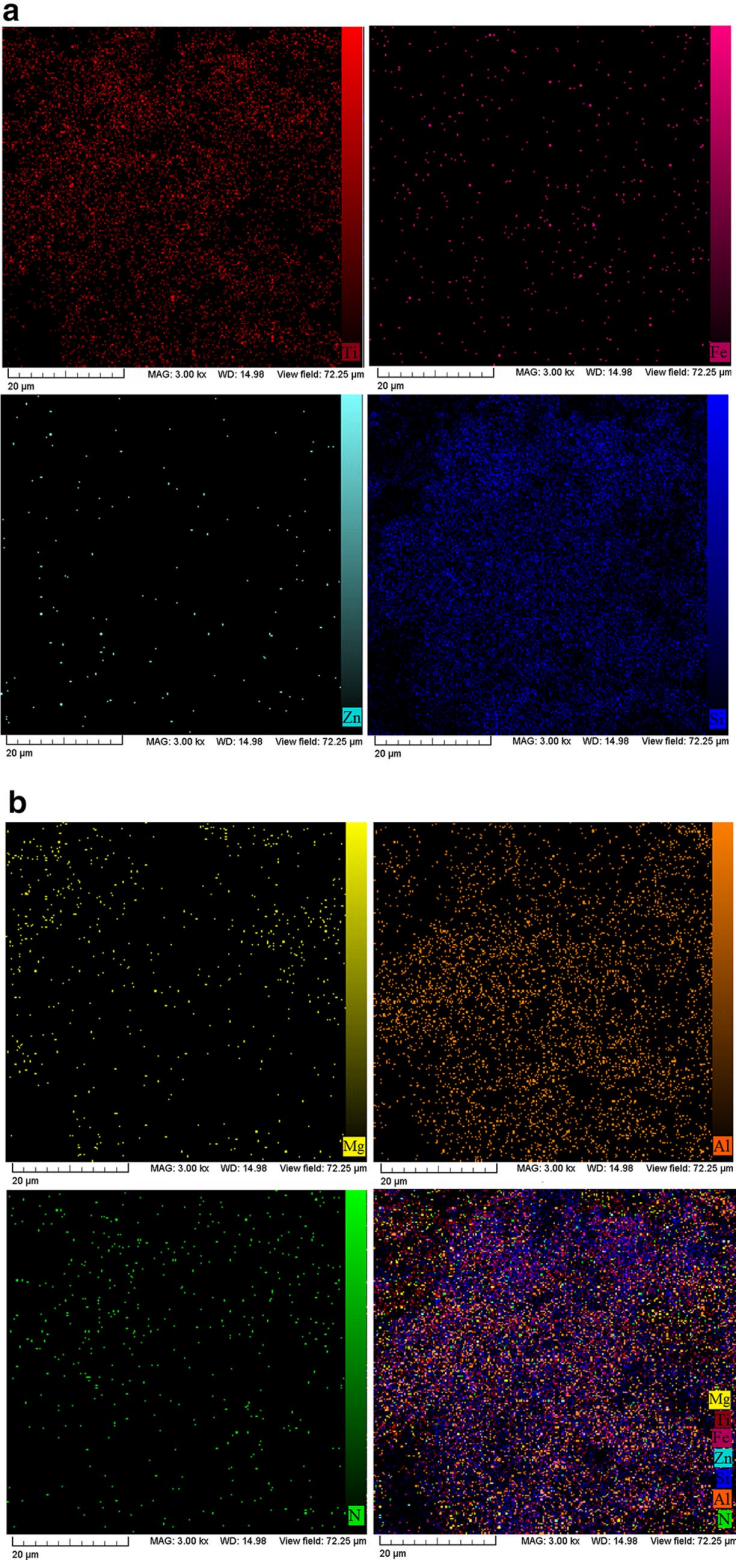
Analysis of bitumen modified with clay/silica nanoparticles (CS-NPs) by means of EDS mapping: elemental spot maps of titanium (Ti), iron (Fe), zinc (Zn), silicon (Si), magnesium (Mg), aluminium (Al), Nitrogen (N), and combination intensity map of all seven elements.

In order to investigate the distribution of CS-NPs in bitumen, 1 wt.% of titanium dioxide (having 1 µm average particle size) was added to a control bitumen sample modified with CS-NPs, and the distribution of elements was analyzed by Energy Dispersive X-ray (EDX) mapping. The results are illustrated in Fig. 4.

The titanium element map indicates that bitumen distribution is homogeneous. Furthermore, in order to investigate silica distribution, 0.1 wt.% zinc oxide was added to fumed silica NPs. Based on zinc energy dispersive X-ray spectrometry (EDS) mapping^34^, it was found that fumed silica NPs coated clay layers uniformly. In addition, zinc dispersion indicates that this technique of producing hydrothermal CS-NPs is suitable for modified homogenous bitumen blending. The EDS map of nitrogen refers to the nitrous oxide in silica NPs, and also could be an indicator of nitrogen elements in bitumen. The dispersion of clay composing elements (silica, aluminum, and iron) on the bitumen surface was also verified by EDS mapping, where, silicon, aluminium, and iron were found to be distributed uniformly on the surface, representing a suitable saturation of CS-NPs in bitumen samples. According to the components of clay identified in previous work^35^, the values of iron oxide and magnesium oxide are approximately the same, which is validated in this study by the iron and magnesium elements maps. Furthermore, the combination map confirms the successful coating of fumed silica NPs on clay nanolayers.

### Rheological properties identified with DSR

#### Complex modulus and phase angle

Rheological properties of bitumen modified by CS-NPs were studied in dynamic shear rheometer (DSR) based on measurements of complex shear modulus G* and corresponding phase angle δ, both depending on temperature and loading frequency^36,37^.

The bitumen samples were investigated before and after laboratory aging. Laboratory aging was conducted through Rolling Thin Film Oven Test (RTFOT), referred to as short term aging procedure according to the US Standard (ASTM D1754), and through exposure to UV radiation for durations of 6 days and of 12 days.

**Figure 5:**
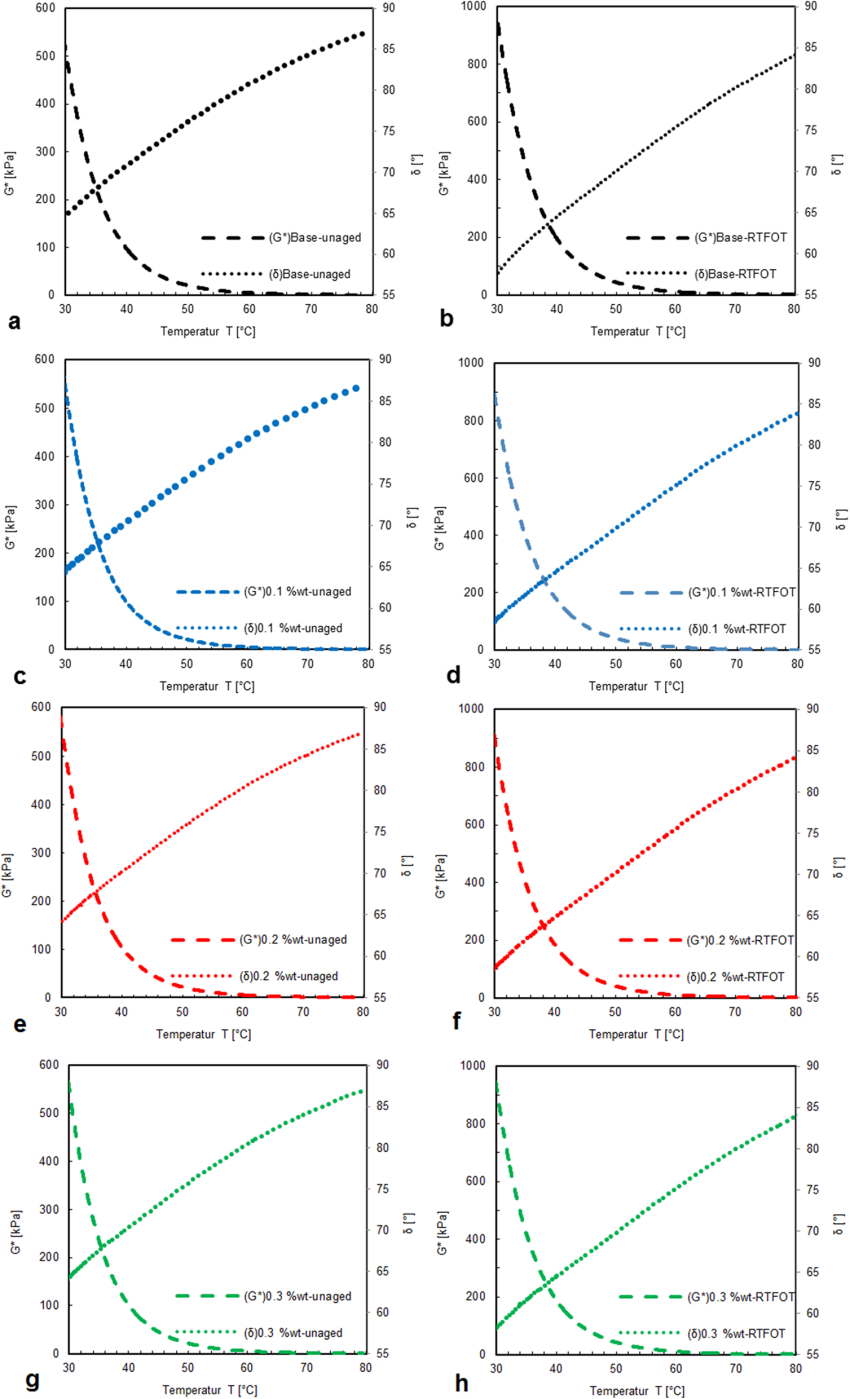
Complex modulus and phase angle of modified bitumen with CS-NPs before laboratory aging (**a**, **c**, **e**, **g**) and after (**b**, **d**, **f**, **h**) short-term aging in the Rolling Thin Film Oven Test (RTFOT), with different concentrations: without CS-NPs (**a**, **b**), with 0.1 wt.% CS-NPs (**c**, **d**), with 0.2 wt.% CS-NPs (**e**, **f**), and with 0.3 wt.% CS-NPs (**e**, **g**).

The results representing a temperature range from 30 to 80 °C are shown in Fig. 5.

The level of complex shear modulus was found to be significantly higher for bitumen samples modified with CS-NPs than for unmodified control bitumen samples. Hence, the addition of 0.2 wt.% of CS-NPs increases stiffness (and thus deformation resistance of the corresponding asphalt mixture). Ranking of samples before aging was

- for complex shear modulus G*:
  without CS-NPs < with 0.1 wt.% of CS-NPs < with 0.3 wt.% of CS-NPs < with 0.2 wt.% of CS-NPs,
- and for phase angle δ:
  with 0.2 wt.% of CS-NPs > without CS-NPs > with 0.1 wt.% of CS-NPs > with 0.3 wt.% of CS-NPs.

Because of laboratory aging, stiffness levels are increased, and phase angles are decreased. This goes together with a distinct increase in the bitumens’ elastic behavior^23^. According to Fig. 5, the bitumen sample including 0.2 wt.% CS-NPs shows highest stiffness in short-term aging.

**Figure 6:**
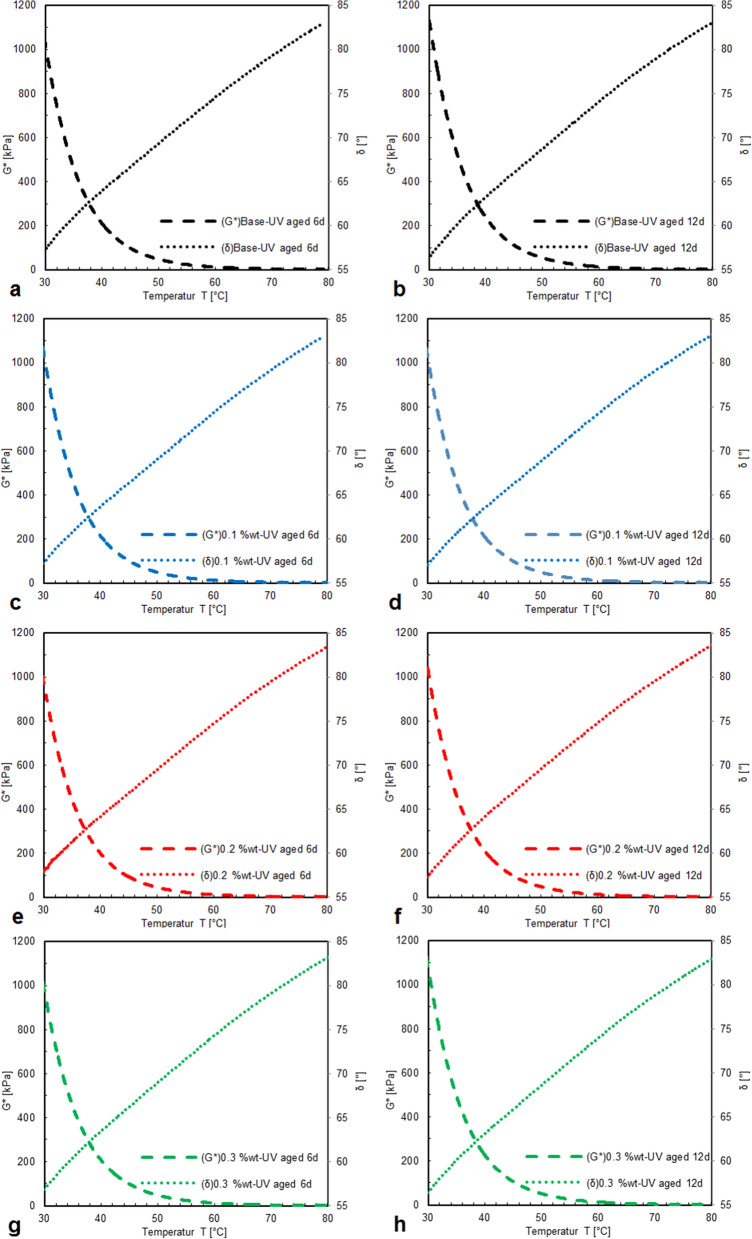
Complex shear modulus G* and phase angle δ of bitumen modified with CS-NPs after UV aging of 6 days (**a**, **c**, **e**, **g**) and 12 days (**b**, **d**, **f**, **h**); bitumen samples of different concentrations: without CS-NPs (**a**, **b**), with 0.1 wt.% of CS-NPs (**c**, **d**), with 0.2 wt.% of CS-NPs (**e**, **f**), and with 0.3 wt.% of CS-NPs (**e**, **g**).

In Fig. 6, the results UV aging are illustrated, indicating that UV aging increases complex shear modulus of the unmodified control bitumen. Remarkably, for bitumen modified with CS-NPs the aging effect is reversed. The ranking of complex shear modulus and of phase angle in 6 days of UV aging was found to be.

- for complex shear modulus G*:
  with 1.0 wt.% of CS-NPs > without CS-NPs > with 3.0 wt.% of CS-NPs > with 2.0 wt.% of CS-NPs,
- and for phase angle δ:

with 3.0 wt.% of CS-NPs < with 1.0 wt.% of CS-NPs < with 1.0 wt.% of CS-NPs < without CS-NPs.

Maximum and minimum values of complex shear modulus and phase angle in 12 days of UV aging are observed for the sample with 0.2 wt.% CS-NPs, and the sample with 0.1 wt.% CS-NPs. The sample with 0.2 wt.% of CS-NPs is assumed to be most UV resistant, as an optimum percent of CS-NPs can be considered most efficient to shield the bitumen from UV radiation.

#### Resistance to permanent deformation

**Figure 7:**
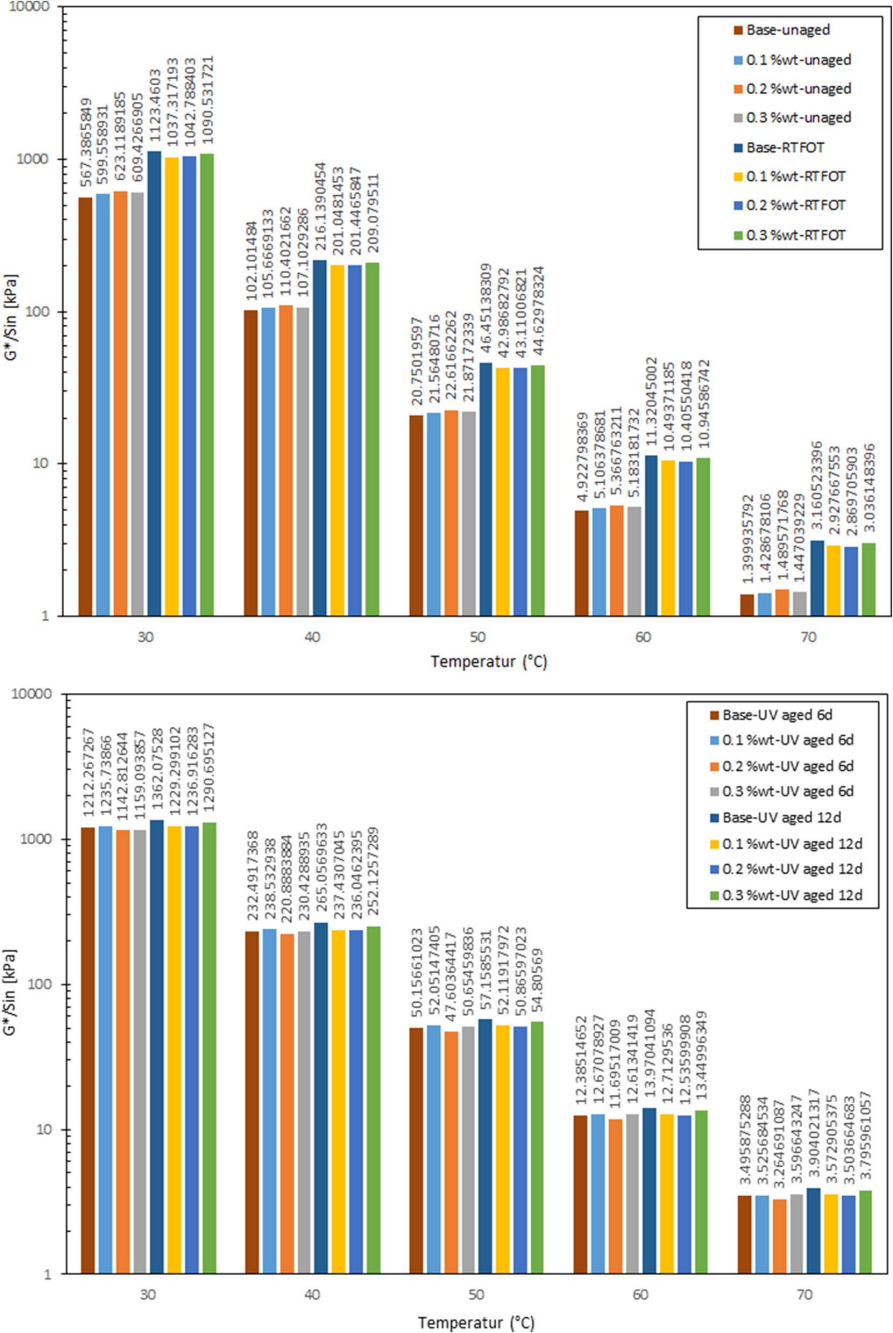
Rutting resistance of bitumen modified with CS-NPs in unaged condition, in short-term aged condition (RTFOT), and in UV aged condition.

Figure 7 displays permanent deformation resistance of bitumen samples between temperatures of 30 °C and 70 °C, before and after laboratory aging. The rutting factor indicates rutting resistance or stiffness of bitumen at high temperature. Based on the US-Standard AASHTO T 315, complex modulus and phase angle at 10 rad/s were selected for calculating the rutting factor^37^.

After short-term aging (through RTFOT), the rutting factor increases for the unmodified control bitumen, while for the bitumen samples modified with CS-NPs the rutting factor decreases. Hence, a better resistance to permanent deformation can be assumed for the bitumen samples modified with CS-NPs.

The results also show that the influence of short-term aging on the resistance to permanent deformation is more dominant than the influence of UV aging. Nevertheless, the effect of UV aging is undeniable. During UV aging, the bitumen sample modified with 0.3 wt.% of CS-NPs is less changed, than the control bitumen sample, while other concentrations of NPs have remarkable effects on the resistance to permanent deformation. As highlighted in Fig. 7, the value of the bitumen sample modified with 0.2 wt.% of CS-NPs is the smallest, which indicates that CS-NPs cause a high resistance to UV radiation.

In this study, rutting resistance is judged based on the threshold temperatures derived from the performance grade approach as proposed by SHRP Standard (SHRP-A-369)^38–40^. Based on the results represented in Table 1, the temperatures at which G*/sinδ equals 1 kPa, and 2.2 kPa respectively, are used as threshold temperatures before and after aging (according to SHRP standard). These results indicate that the CS-NPs decreased the rutting resistance of the bitumen sample before aging. During aging, the threshold temperatures of the control bitumen samples were increased, and thus, the bitumen samples became harder. Addition of CS-NPs decreased the threshold temperature.

**Table 1:** Bitumen threshold temperatures in different concentrations of CS-NPs before and after aging.

Threshold temperatures (°C)															
Before aging (G*/sin δ = 1 kPa)				After aging (G*/sin δ = 2.2 kPa)											
S1	S2	S3	S4	S5	S6	S7	S8s	S9	S10	S11	S12	S13	S14	S15	S16
72.8	73.1	73.4	73.1	73.0	72.4	72.9	72.7	73.9	73.9	73.3	74.2	74.8	74.1	73.9	74.6

From these results it can be concluded that the bitumen modification with CS-NPs reduces the stiffness increase of bitumen samples subjected to UV aging and retards bitumen hardening in consequence of aging. Moreover, the results show that the addition of 0.1 and 0.2 wt.% of CS-NPs results in preferable performance after short-term aging and UV aging than higher concentrations.

### Interaction of bitumen and nanoparticles

In order to investigate potential reasons for the anti-aging effects of bitumen with nanoparticles, the molecules' interactions need to be considered. Initial interaction is related to increasing hydroxy groups bondings between bitumen and silica NPs surfaces. This subject is due to the high surface energy of silica nanoparticles^41,42^. In addition, the high surface area to volume ratio of clay nanolayers is a significant factor which increases the ability of bitumen molecules to interact with clay layers^11^. In other words, silica NPs are connected with bitumen molecules by chemical bonds, and bitumen components interact with NPs by means of physical reactions (van der Waals forces)^43^.

**Figure 8:**
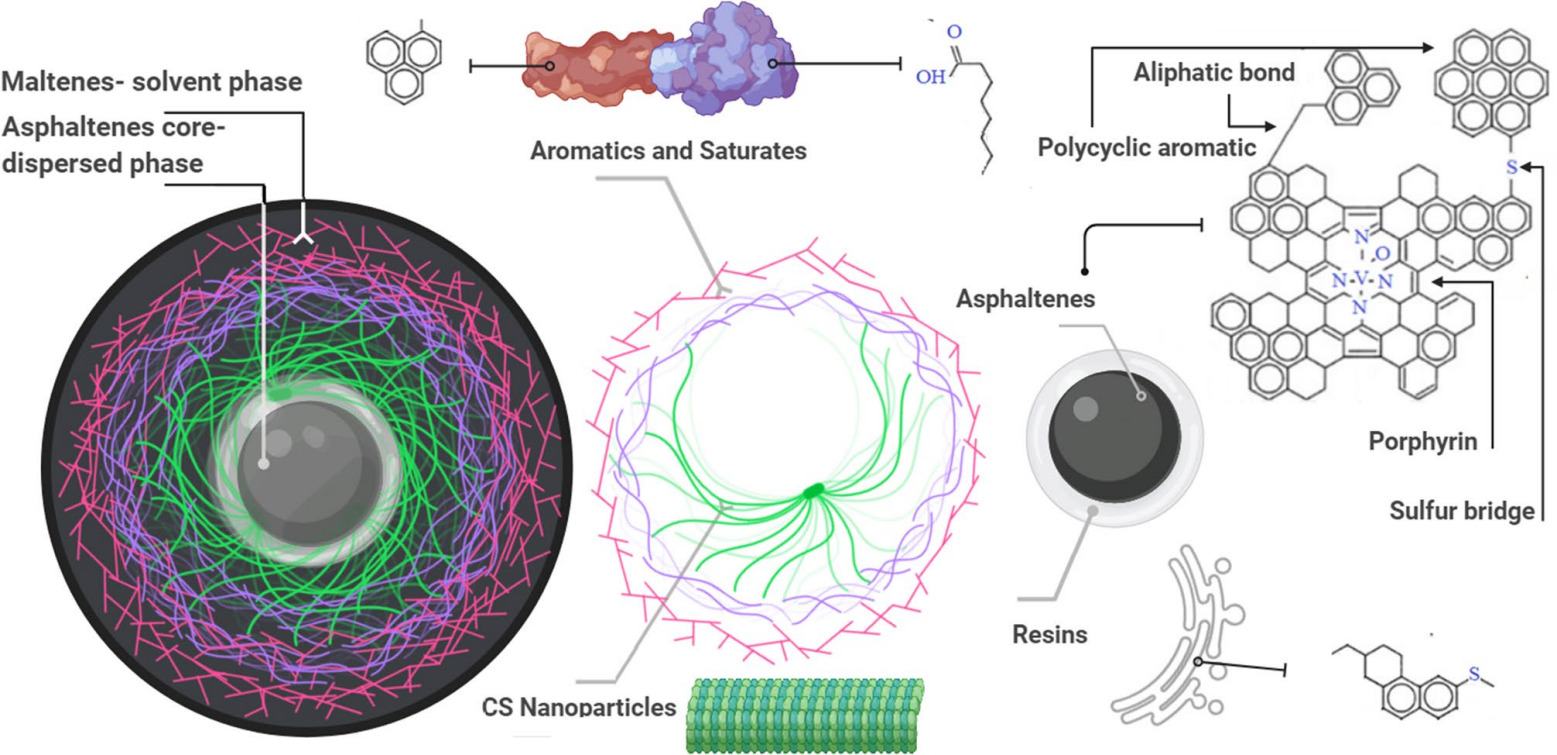
Schematic illustration of the CS-NPs action in the colloidal structure of bitumen.

Based on the colloidal structure of bitumen (Fig. 8), asphaltenes are the dispersed phase in the solvent phase. The average diameter of asphaltenes is 0.5–40 nm^44,45^, and therefore they can create significant changes in material nature. CS-NPs (average particle sizes of clay NPs and silica NPs are about 12 and 33 nm, respectively) may chemically react and disperse between these colloidal dimensions (schematically shown in Fig. 8).

Furthermore, nanolayers of clay change the surface characteristics of bitumen. The polarity of clay layers (related to some elements) decreases the polarity and adsorption of asphaltenes. This is a complex mechanism in which sodium elements (which exist in clay) adsorbed by asphaltene carbons cause saturate molecules.

They disperse in the colloidal structure of bitumen with nanolayers of 1–100 nm in scale, which hinders the penetration of oxygen to the bitumen^46^. The nanoparticles also increase the stability of modified bitumen and avoid destruction of the chemical structure of bitumen components. However, excessive use of clay NPs may destroy the elastic properties of bitumen^47^. Note that there are most probably other chemical and physical properties of these nanoparticles in addition, such as ion exchange reactions and wettability alteration^48^.

### Chemical composition identified with FT-IR

**Figure 9:**
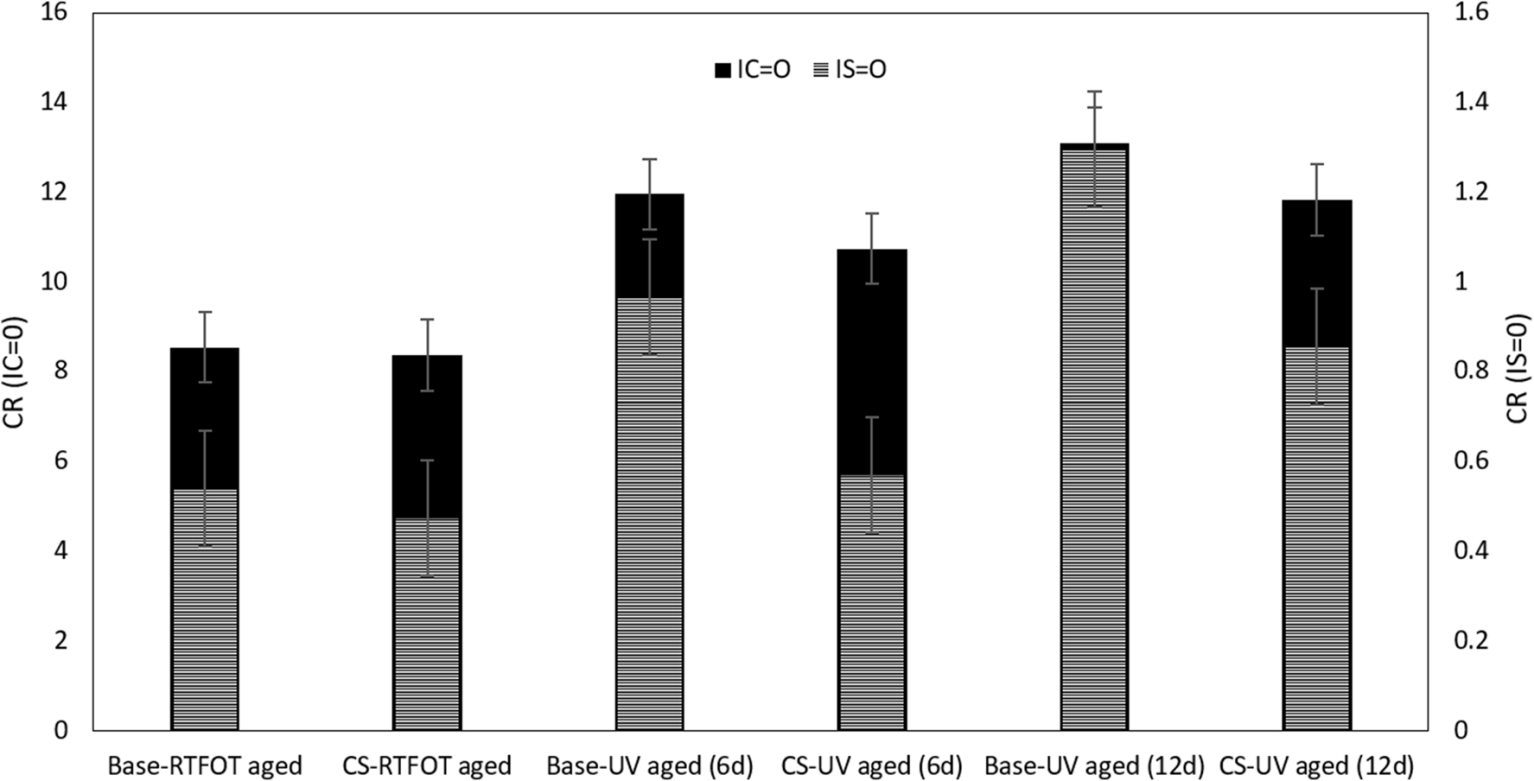
Aging indexes based on carbonyl and sulfoxide groups for different aging conditions.

Figure 9 presents the change rate (CR) of the carbonyl and sulfoxide index for short term and for UV aging. The carbonyl index increased more for UV aged samples than for control bitumen samples. The carbonyl and sulfoxide index is decreased for bitumen samples modified with CS-NPs. Furthermore, carbonyl and sulfoxide index increased with expanding aging time.

Figure 9 shows the difference in CR when carbonyl is increased to 3.41, and to 1.08 respectively, and in sulfoxide index when sulfoxide is increased to 0.426, and to 0.285 respectively, 6 and 12 days of UV radiation, without and with NPs. These data indicate that the use of CS-NPs results in suitable performance due to their efficient UV-shielding coating. It can be assumed, that NPs with nano-diameter have an excellent capability to absorb UV light^49^, and in addition, clay nano layer is a suitable material in reflecting UV light. The results indicate that increasing the content of NPs by more than 0.2 wt.% will increase UV aging resistance significantly. However, the optimum content depends on many factors, such as type, size and solvent of NPs in mortar and in bitumen^50^.

### Mechanical properties

For validation of FTIR results, the index of viscosity aging (IVA) and softening point increment (SPI) are used^31^. IVA and SPI are computed from Eqs. (1) and (2):

1 $${\text{ IVA}} = \frac{{{\text{viscosity }}\;{\text{of }}\;{\text{bitumen }}\;{\text{after }}\;{\text{aging}} - {\text{viscosity }}\;{\text{of }}\;{\text{unaged }}\;{\text{bitumen}}}}{{{\text{viscosity }}\;{\text{of }}\;{\text{unaged}}\;{\text{ bitumen}}}}$$

2 $${\text{SPI}} = {\text{softening}}\;{\text{ point}}\;{\text{ of}}\;{\text{ aged}}\;{\text{ bitumen }}{-}{\text{ softening }}\;{\text{point }}\;{\text{of }}\;{\text{unaged }}\;{\text{bitumen}}$$

**Figure 10:**
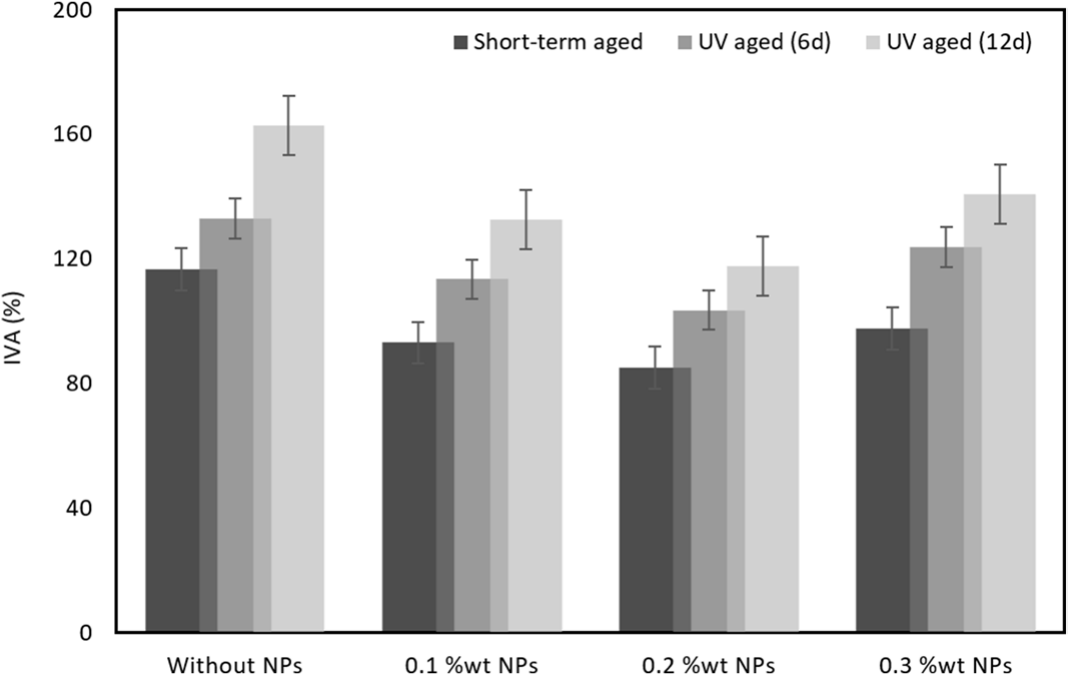
Index of viscosity aging (IVA) of control bitumen and of bitumen modified with CS-NPs.

**Figure 11:**
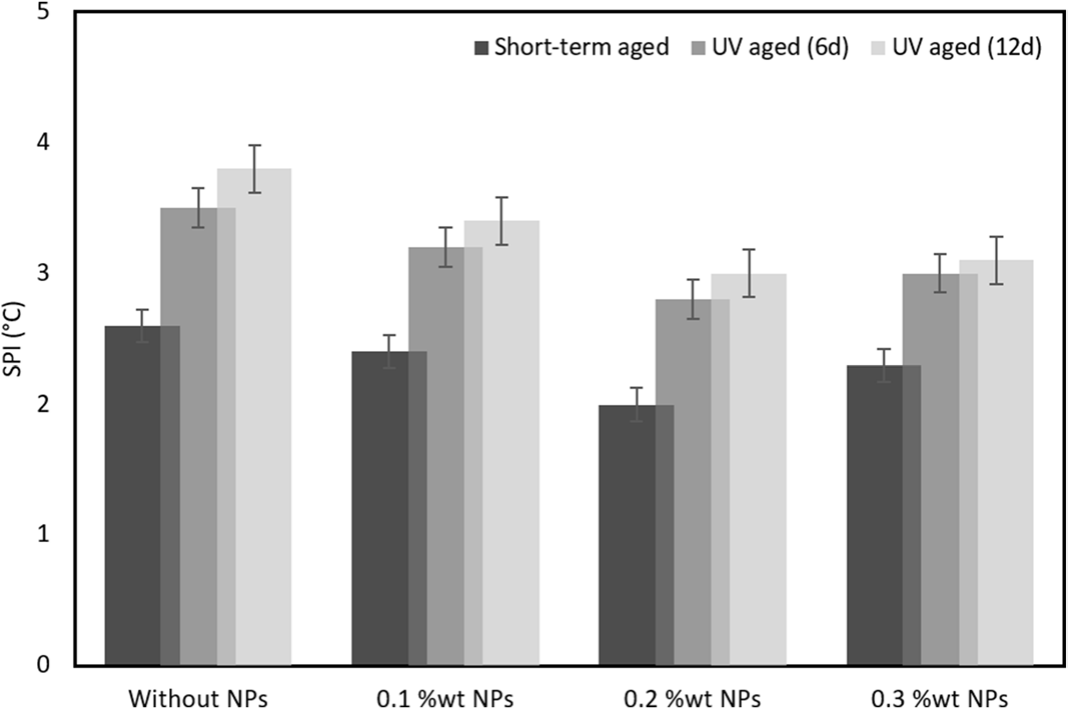
Softening point increment (SPI) of control bitumen and of aged bitumen modified with CS-NPs.

After short term aging and UV aging, IVA decreases for samples modified by CS-NPs, which confirms the findings stated earlier (Fig. 10). IVA is increased, when the duration of UV aging is increased. This can be seen in Fig. 10, where smallest level of IVAs are found for samples S11 and S15, 6 and 12 days of UV aging. Moreover, an increase of the concentration of fumed silica nanoparticles in bitumen goes together with an increase in IVA and a reduction in aging resistance. SPI values for bitumen samples with different contents of CS-NPs are shown in Fig. 11. These results illustrate that the effect of CS-NPs is more distinct for UV aging than for short term aging.

## Conclusions

Surface morphology, rheological and chemical properties were investigated of bitumen, which was modified by a composite of clay and fumed silica nanoparticles, and exposed to ultraviolet (UV) aging in laboratory. The volume fraction of the nanoparticles within the binder ranged from 1 to 3%, the temperature range considered was 30 to 70 °C. Surface morphology, rheological and chemical binder properties were analyzed using field emission scanning electron microscopy (FESEM), dynamic shear rheometer (DSR), and Fourier transform infrared (FT-IR) spectroscopy. From these investigations, the following main conclusions can be drawn:

The bitumen modification through clay and fumed silica nanoparticles changed binder properties significantly. The distinct surface of clay nanolayers and the high UV reflectivity of fumed silica NPs caused a change in bitumen performance. The index of carbonyl and oxidation degree decreased, and the clay and fumed silica nanoparticles improved aging resistance to ultraviolet (UV) radiation considerably, as shown by FTIR results. It is concluded that clay and fumed silica nanoparticles may potentially be used as efficient UV-shielding coatings in asphalt pavements.

Adding clay and fumed silica nanoparticles to bitumen reduced stiffness distinctly, and improved resistance to permanent deformation. The results indicate that the mechanical stability of the modified bitumen is very much driven by the specific concentration of clay and fumed silica nanoparticles. The DSR and FT-IR results indicate that increasing the content of NPs by more than 0.2 wt.% will increase UV aging resistance significantly. Moreover, deformation resistance shows the bitumen modification with CS-NPs reduces the stiffness increase of bitumen samples subjected to UV aging and retards bitumen hardening in consequence of aging.

Bitumen modification through clay and fumed silica nanoparticles can be considered as an interesting low-cost technique in asphalt pavement engineering providing novel perspectives in making asphalt materials more durable. From a broader perspective, our findings of molecular interactions between nanoparticles and bitumen will open up a new avenue that will be an inspiration in the nanotechnology concept in asphalt.

## Methods

### Materials and synthesis

In the present research, sodium bentonite (Sigma Aldrich Ltd., Germany), nano fumed silica (Aerosil A300, Degussa Co., Germany), and 50/70 penetration grade bitumen (Total Co., France) were used. The montmorillonite chemical composition is reported as follows: 61.03% SiO_2_, 14.59% Al_2_O_3_, 2.22% MgO, 0.22% TiO_2_, 2.09% Fe_2_O_3_, 2.04% Na_2_O, 0.76% K_2_O and 0.77% CaO.

**Figure 12:**
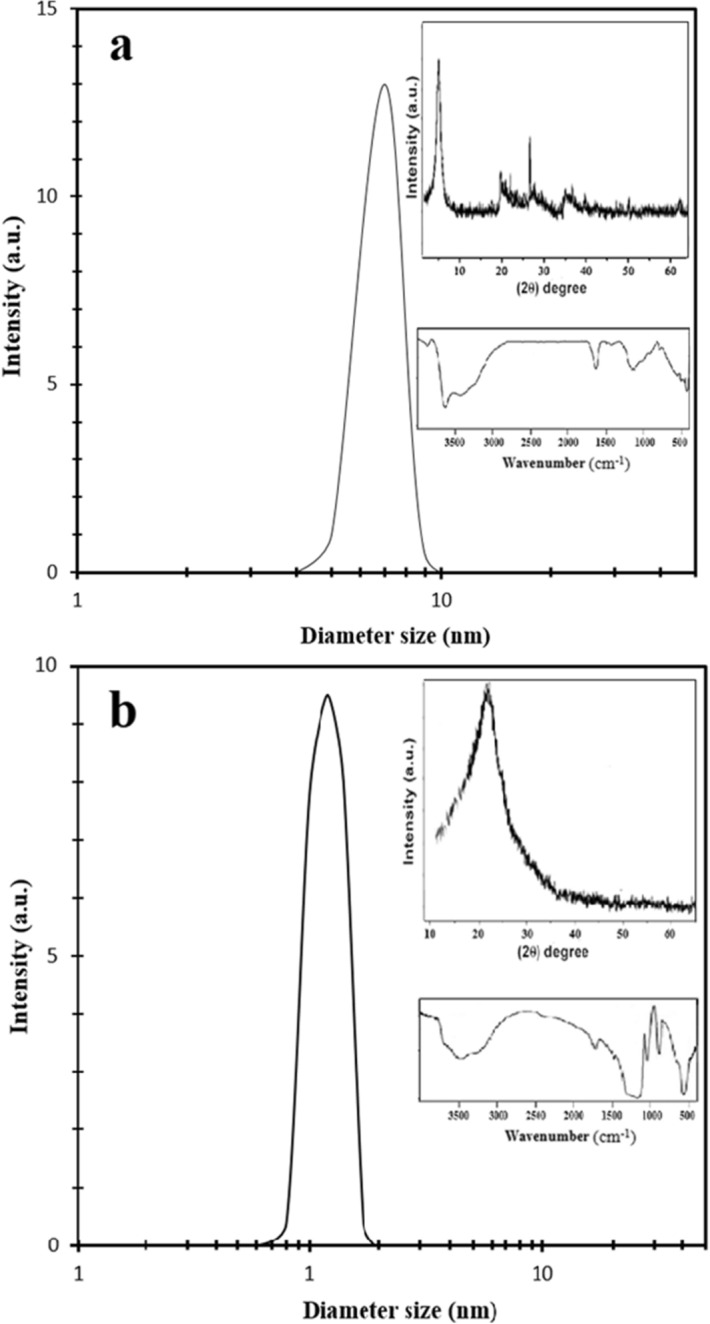
DLS distribution, XRD pattern, and Fourier transform infrared spectra of (**a**) clay; and (**b**) fumed silica.

**Figure 13:**
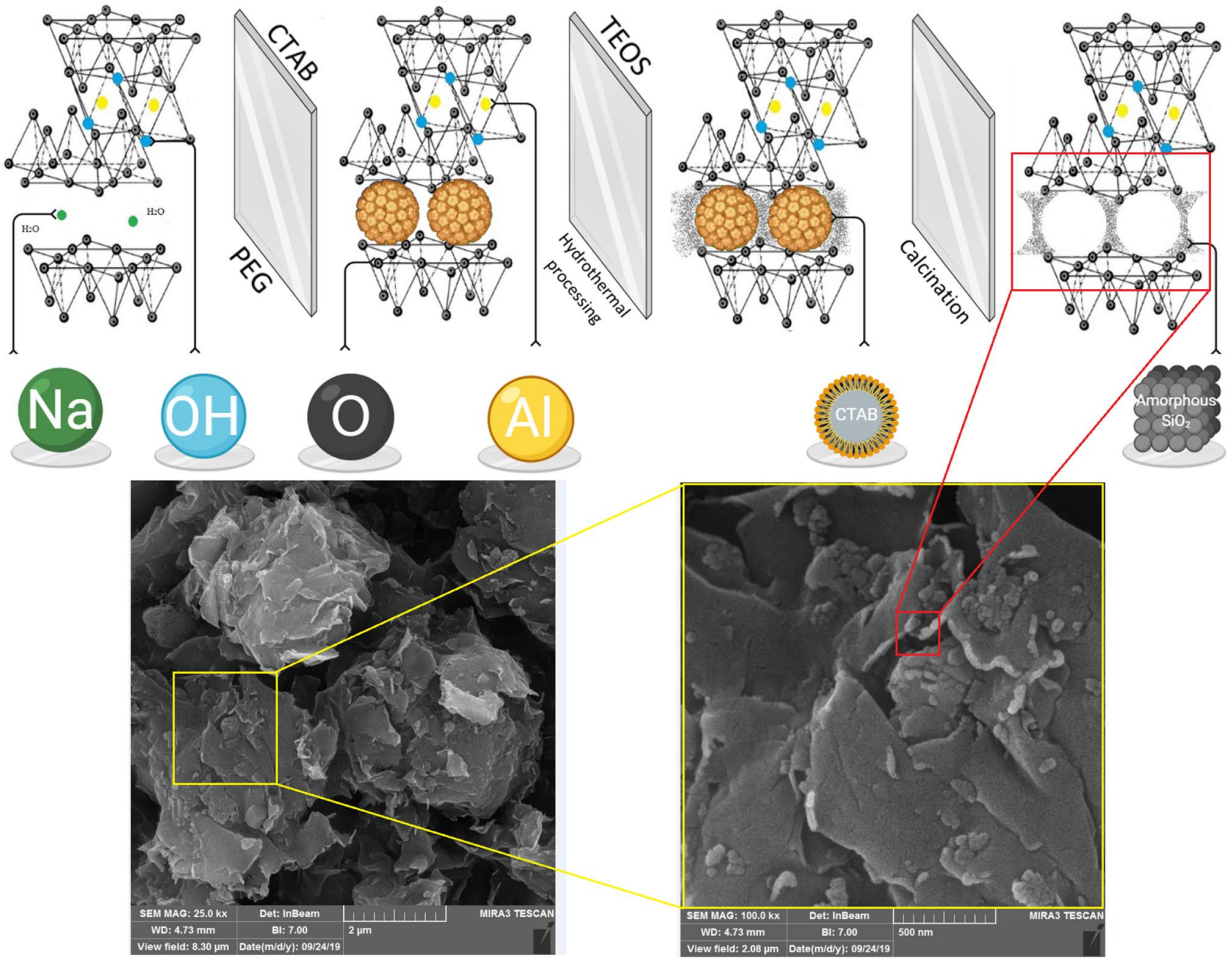
Schematic illustration of multistep synthesis of CS-NPs.

Clay/silica was prepared with hydrothermal syntheses method using a procedure adapted from Yang et al.^51^ and Cheraghian et al.^30,52^. The size distribution of materials and X-ray diffraction (XRD) pattern were evaluated with dynamic light scattering (DLS) (Malvern ZEN 3600, UK) and X-ray powder diffraction (Philips PW 1730, Netherlands) analyses, as shown in Fig. 12. After purification of montmorillonite, it was milled to exchange 98 mmol/100 g cation capability. Then, 4 g of montmorillonite and 0.48 g of NaOH were dissolved for 3 h within 200 ml deionized water at a temperature of 25 °C, and thereupon, they were dispersed by ultrasonic mixer. Then, 2 ml of polyethyleneglycol (PEG) and 4 g of cetyltrimethylammoniumbromid (CTAB) were dispersed in 40 ml of distilled water. PEG/CTAB solution was blended to the mixture for 3 h, and at the same time 10 ml of tetraethylorthosilicate (TEOS) was injected to the suspension. The mixed materials in a stainless-steel autoclave were heated to a temperature of 180 °C for 16 h, and then that the materials were cooled down to 25 °C. Finally, the powders were heated to eliminate the organic surfactant. Figure 13 indicates the schematic synthesis process of clay and fumed silica.

### Ageing process

For Rolling Thin Film Oven Test (RTFOT), thin films of bitumen samples were exposed to airstream of 163 °C (RTFOT 8, ISL Product, France) according to ASTM D1754.

For UV aging, the bitumen samples were put in shells of 90 ± 0.5 mm in diameter, which were exposed to UV radiation of a 500 W lamp for durations of 6 and 12 days at 50 °C with average intensity of 10 W/m^2^.

Bitumen samples were prepared in three conditions: samples unaged (S1–S4), samples aged in RTFO conditions (S5–S8), and samples aged in UV conditions (S9–S16). All samples are presented in Table 2.

**Table 2:** Overview of sample aging processes.

Sample no.	Content of CS-NPs (wt.%)	Ageing process	Sample no.	Content of CS-NPs (wt.%)	Aging process
S1	–	Unaged	S9	–	6d UV
S2	0.1	Unaged	S10	0.1	6d UV
S3	0.2	Unaged	S11	0.2	6d UV
S4	0.3	Unaged	S12	0.3	6d UV
S5	–	RTFO	S13	–	12d UV
S6	0.1	RTFO	S14	0.1	12d UV
S7	0.2	RTFO	S15	0.2	12d UV
S8	0.3	RTFO	S16	0.3	12d UV

### Characterization methods

#### Fourier-transform infrared spectroscopy test (FTIR)

Samples were tested through FTIR in transmission mode, between 400 to 4,000 cm^−1^ spectra range (Nexus, Thermo Nicolet Corp., USA). Chemical structure of materials can be determined with range of spectra in different chemical bands. The FTIR detects the reflection of the various infrared spectrum of chemical bonds. Carbonyl and Sulfoxide bonds result from UV radiation process, which forms carbon–carbon or carbon–hydrogen bonds. Carbonyl (C=O) and sulfoxide (S=O) functions were monitored with spectra of 1,700 cm^−1^, and of 1,030 cm^−1^ respectively. Both values can be determined from the range of oxidation in asphalt. C=O group index (IC=O) and S=O group index (IS=O) can be calculated by Eqs. (3) and (4):

3 $${\text{I}}_{{\text{C } = \text{ O}}} = \frac{{{\text{Area}}\;{\text{ of }}\;{\text{carbonyl }}\;{\text{band }}\;{\text{centered }}\;{\text{around}}\; 1700 \;{\text{cm}}^{ - 1} }}{{\sum {\text{Area }}\;{\text{of }}\;{\text{spectral }}\;{\text{bands}}\;{\text{ around}} \;1460 \;{\text{and}}\; 1375\; {\text{cm}}^{ - 1} }}$$

4 $${\text{I}}_{{\text{S } = \text{ O}}} = \frac{{{\text{Area }}\;{\text{of }}\;{\text{carbonyl }}\;{\text{band}}\;{\text{ centered}}\;{\text{ around}} \;1030 \;{\text{cm}}^{ - 1} }}{{\sum {\text{Area}}\;{\text{ of}}\;{\text{ spectral}}\;{\text{ bands }}\;{\text{around}}\; 1460 \;{\text{and}}\; 1375 \;{\text{cm}}^{ - 1} }}$$

5 $${\text{ CR}} = \frac{{{\text{Index }}\;{\text{of }}\;{\text{bitumen }}\;{\text{after}} - {\text{Index }}\;{\text{of }}\;{\text{bitumen }}\;{\text{before}}}}{{{\text{Index}}\;{\text{ of }}\;{\text{bitumen}}\;{\text{ before}}}}$$

#### Rheological testing (DSR)

A dynamic shear rheometer (DSR) (Malvern Kinexus Pro+, UK) was used to evaluate binder rheological properties in the domain of linear viscoelastic behavior, under different conditions (temperatures between 20 and 70 °C, and frequency of 10 rad/s). The complex shear modulus (G*), phase angle (δ), and rutting factor (G*/sinδ) of control bitumen and aged bitumen samples were investigated, based on the US-Standard AASHTO T 315.

#### Scanning electron microscope (SEM)

A FE-SEM (TE-SCAN, MIRA III, Czech Republic) was used to consider and validate micro and nano structures of additives in bitumen samples. The morphology was characterized by focusing an electron beam on the sample surface. In order to prevent electron surface charging during imaging, the surface of sample was coated with a thin film of gold (10 nm film thickness) before FE-SEM imaging.

#### Mechanical tests

A Petrotest machine was used for identifying the bitumens’ softening point ring and ball (PKA5, Germany) with standard ASTM D36. Ductility test (Infratest, 20-2356, Germany) was performed with a ductilometer machine 1,500 mm digital, according to Standard ASTM D113. An automatic penetrometer from Anton Paar (PNR 12, Germany) was used to determine Needle Penetration according to Standard ASTM D5. Table 3 summarizes the physical properties of bitumen determined in this study.

**Table 3:** Physical properties of bitumen sample.

Physical properties	Value	Standard
Ductility (@25°C, cm)	100	ASTM D113
Softening point (°C)	48.6	ASTM D36
Penetration (@25°C, 0.1 mm)	63	ASTM D5
Density (kg/m^3^)	1.03	ASTM D70
